# Molecular Mechanisms Behind Anti SARS-CoV-2 Action of Lactoferrin

**DOI:** 10.3389/fmolb.2021.607443

**Published:** 2021-02-15

**Authors:** Mattia Miotto, Lorenzo Di Rienzo, Leonardo Bò, Alberto Boffi, Giancarlo Ruocco, Edoardo Milanetti

**Affiliations:** ^1^ Department of Physics, University of Rome `La Sapienza', Rome, Italy; ^2^ Istituto Italiano di Tecnologia (IIT), Center for Life Nano Science, Rome, Italy; ^3^ Department of Biochemical Sciences “A. Rossi Fanelli” Sapienza University, Rome, Italy

**Keywords:** lactoferrin, SARS-CoV-2, shape complementarity, spike (S) glycoprotein, sialic acid, molecular dynamics simulation

## Abstract

Despite the huge effort to contain the infection, the novel SARS-CoV-2 coronavirus has rapidly become pandemic, mainly due to its extremely high human-to-human transmission capability, and a surprisingly high viral charge of symptom-less people. While the seek for a vaccine is still ongoing, promising results have been obtained with antiviral compounds. In particular, lactoferrin is regarded to have beneficial effects both in preventing and soothing the infection. Here, we explore the possible molecular mechanisms with which lactoferrin interferes with SARS-CoV-2 cell invasion, preventing attachment and/or entry of the virus. To this aim, we search for possible interactions lactoferrin may have with virus structural proteins and host receptors. Representing the molecular iso-electron surface of proteins in terms of 2D-Zernike descriptors, we 1) identified putative regions on the lactoferrin surface able to bind sialic acid present on the host cell membrane, sheltering the cell from the virus attachment; 2) showed that no significant shape complementarity is present between lactoferrin and the ACE2 receptor, while 3) two high complementarity regions are found on the N- and C-terminal domains of the SARS-CoV-2 spike protein, hinting at a possible competition between lactoferrin and ACE2 for the binding to the spike protein.

## Introduction

Lactoferrin (Lf) is a versatile glycoprotein, which plays a key role in many biological functions Redwan et al. (2014). In this work, we focus on the Lf as a crucial player in natural immunity, since it has been proposed to play a strong antiviral activity against a wide range of RNA and DNA viruses Puddu et al. (1998), Andersen et al. (2001), Drobni et al. (2004), Marchetti et al. (2004), and Waarts et al. (2005). Lf is composed of a single chain of about 700 residues folded into two symmetrical lobes. Each lobe possesses a metal-binding site, able to bind iron but also other ions like $Cu^{2+}$, $Zn^{2+}$, and $Mn^{3+}$ Baker and Baker (2005) and Giansanti et al. (2016).

This protein is present in saliva, tears, seminal fluid, white blood cells, and milk of mammals Niaz et al. (2019). From its discovery in 1939 Sorensen and Sorensen (1940) and Groves (1960) lactoferrin has been identified as the most important iron-binding protein in milk. Besides, in recent years, lactoferrin has been found involved in a multitude of biological processes. In fact, despite the name, the iron cargo capacity of Lf is not the prominent activity exerted by this molecule. Instead, it performs antioxidant, anti-inflammatory, and anticancer activities Caccavo et al. (2002) and Giansanti et al. (2016), together with a broad antimicrobial action against bacteria and fungi. The latter activity, in particular, is due to Lf’s ability to reversibly bind two atoms of iron with high affinity in the presence of bicarbonate. The iron-free form of Lf, apo-lactoferrin (apoLf), deprives bacteria of iron, thus inhibiting their metabolic activities in vivo.

Besides all the aforementioned activities, Lf has been demonstrated to prevent infection of a wide range of diverse viral species van der Strate et al. (2001).

Many viruses make use of glycans as attachment factors. Such glycans are chain-like structures composed of sugar molecules that decorate the surface of cellular constituents, like membrane proteins Varki (2016) and are involved in many cell functions like support, signaling, protein folding, and protection Bishop and Gagneux (2007) and Miotto et al. (2020).

Two important classes of glycans are sialosides (SIA), which contain sialic acid (SA), and glycosaminoglycans, like heparan sulfate (HF). See, for example, Langford-Smith et al. (2015) for more details. When the contact between the virus particle and these receptors is established, the viruses roll toward their specific viral receptor and subsequently enter the host cell, for instance by fusing with the host cell membrane Raman et al. (2016).

While the interaction between Lf and HF has been observed Lang et al. (2011), studies on its interaction with sialic acid derivatives are still missing. On the other hand, Lf has been reported to interact with virus structural proteins, S, M, and E Yi et al. (1997).

In general, depending on the specifics of the virus, lactoferrin prevents infection of the target cell by either 1) interfering with the attachment factor or 2) by binding to host cell molecules that the virus uses as a receptor or co-receptor (competition) or 3) by direct binding to virus particles, as described for herpesvirus Harmsen et al. (1995), polio- and rotavirus Superti et al. (2001) and McCann et al. (2003) and possibly human immunodeficiency virus Puddu et al. (1998). See, for example, Berlutti et al. (2011) for a more detailed discussion.

While we are writing this article, a novel virus, first observed in the autumn of 2019, has rapidly become pandemic. This virus, called SARS-CoV-2, belongs to the coronavirus family and causes a severe acute respiratory syndrome Huang et al. (2020) and Zhu et al. (2020), somewhat similar to those caused by two other coronaviruses, SARS-CoV and MERS-CoV, which crossed species in 2002–2004 Drosten et al. (2003), Ksiazek et al. (2003), and Zaki et al. (2012). In fact, SARS-CoV-2, similarly to SARS-CoV and MERS-CoV, attacks the lower respiratory system, thus provoking viral pneumonia. However, this infection can also lead to effects on the gastrointestinal system, heart, kidney, liver, and central nervous system Su et al. (2016), Prompetchara et al. (2020), and Zhu et al. (2020).

As for SARS-CoV Li et al. (2005a), Li et al. (2005b), and Li (2008) recent in vivo experiments confirmed that also SARS-CoV-2 cell entry is mediated by high-affinity interactions between the receptor-binding domain (RBD) of the virus S glycoprotein and the human-host Angiotensin-converting enzyme 2 (ACE2) receptor Zhou et al. (2020). The spike protein is located on the virus envelope and promotes the host attachment and fusion between the viral and cellular membrane. Kuo et al. (2000) and Graham and Baric (2010). Structural studies determined the structures of such protein both in free form and bound to ACE2 Yan et al. (2020). Further studies investigate the possible interaction of SARS-CoV-2 to sialic acids Milanetti et al. (2020a), Liu et al. (2020), Robson (2020), and Vandelli et al. (2020), or heparan surfate receptors Liu et al. (2020), both considered involved in SARS-CoV-2 as well as in other coronavirus infections Schwegmann-Weßels and Herrler (2006), Lang et al. (2011), Langford-Smith et al. (2015), Hulswit et al. (2019), and Tortorici et al. (2019).

While the use of Lf as an antiviral against previous coronaviruses infections has been poorly investigated, except for Lang et al. (2011), where evidence of an effect in the attachment process is shown, an increasing number of works hint at the potential antiviral effect of Lactoferrin against the novel SARS-CoV-2 infection Campione et al. (2020), Chang et al. (2020), and de Carvalho et al. (2020).

Here, we computationally investigate the possible molecular mechanisms behind the suggested antiviral action of lactoferrin. In particular, we make use of a recently developed computational protocol based on the 2D Zernike Polynomials, able to rapidly characterize the shape conformation of given protein regions Milanetti et al. (2020a). In this framework using a simple pairwise distance, it is possible to evaluate the similarity between 2 protein pockets or the shape complementarity between the binding regions of 2 interacting proteins.

To assess whether lactoferrin could influence the attachment factors, we investigated the ability of Lt to bind to sialic acid (SIA) or heparan sulfate (HS) receptors Langford-Smith et al. (2015), both considered involved in SARS-CoV-2 infection Milanetti et al. (2020a), Liu et al. (2020), Robson (2020), and Vandelli et al. (2020), as well as to other coronavirus infections Schwegmann-Weßels and Herrler (2006), Lang et al. (2011), Hulswit et al. (2019) and Tortorici et al. (2019).

We moreover checked for a possible direct interaction between LF and ACE2 receptor, which could inhibit the interplay between spike and ACE2 binding necessary to the virus infection.

Finally, we investigate the possible interaction between Lf and the three proteins present on the SARS-CoV-2 membrane, i.e. the spike (S), membrane (M), and envelope (E) proteins.

## Results

**FIGURE 1:**
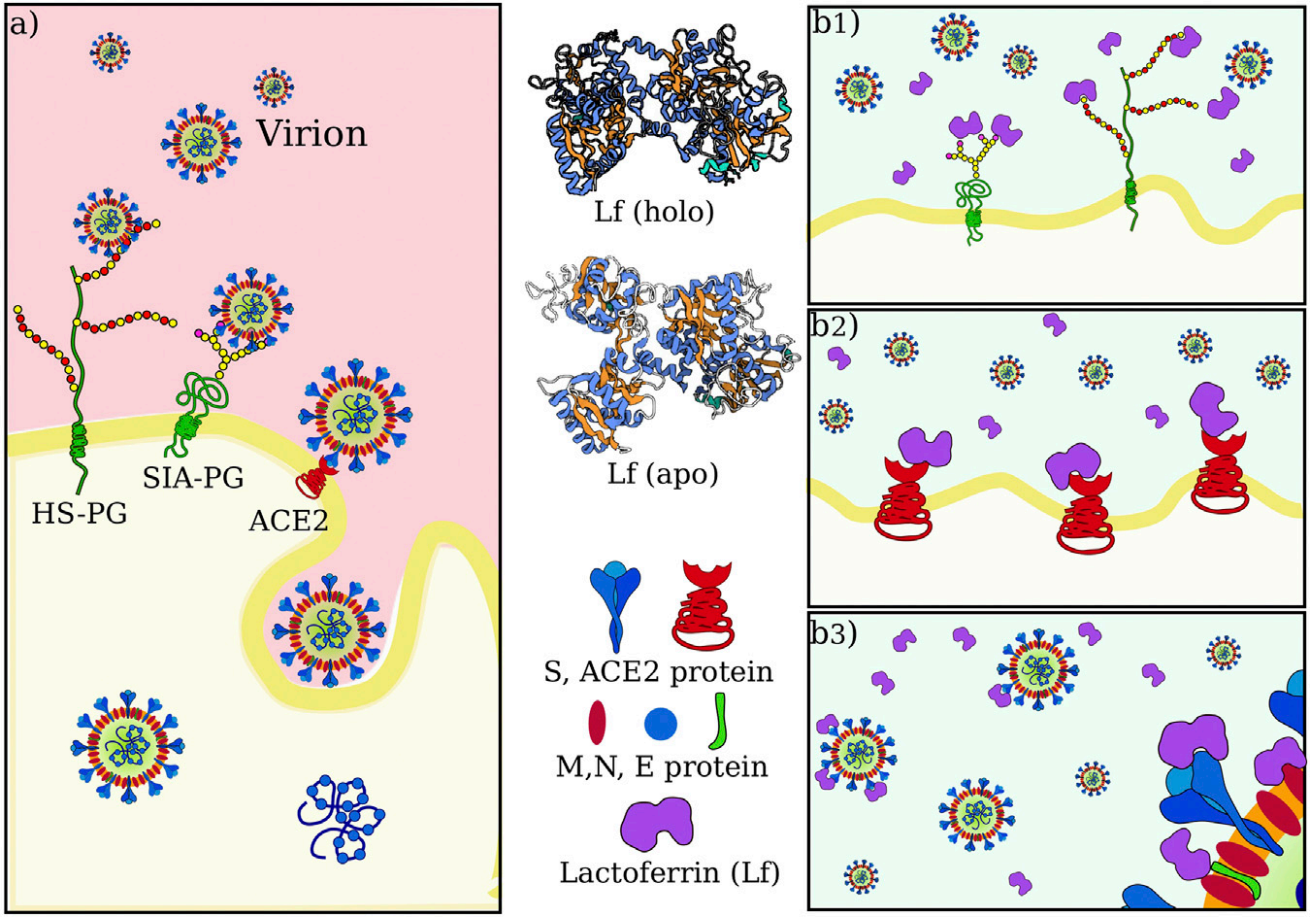
SARS-CoV-2 attachment and entry to host cell in physiological condition and possible actions of lactoferrin. **(A)** Sketch of SARS-CoV-2 initial interactions with the host cell. Sialoside (SIA) and heparan sulfate (HS) glycan chains present on glycoproteins (PG) of the cell membrane are thought to facilitate the attachment of the virion to the cell surface. This favors the establishment of an interaction between the virus spike protein and the ACE2 receptor, which starts the internalization of the virus in the host cell. **(B)** Human lactoferrin has been found to play an antiviral action against SARS-CoV-2 infection although it is not clear whether this action consists in 1) competition for the binding with glycan chains, and/or 2) competition for binding ACE2 receptor and/or 3) direct interaction with one of the proteins in the virion envelope, i.e. with S, M or E proteins.

The entry of the virus inside the host cells requires the occurrence of a sequence of molecular interactions. Sialoside (SIA) and/or heparan sulfate (HF) chains mediate the attachment of the virion to the cell surface. Once in the proximity of the cellular receptor (ACE2), SARS-CoV-2 spike protein binds to the receptor and initiates the internalization process. Figure 1A shows a sketch of the mechanism.

The observed antiviral action of Lf may consist of interference in one or more of those steps. We thus investigate in the next three sections the possibility of direct binding between lactoferrin and sialic acid, ACE2, and SARS-CoV-2 Spike protein, respectively (see Figure 1B).

### Interaction with Sialic Acid

**FIGURE 2:**
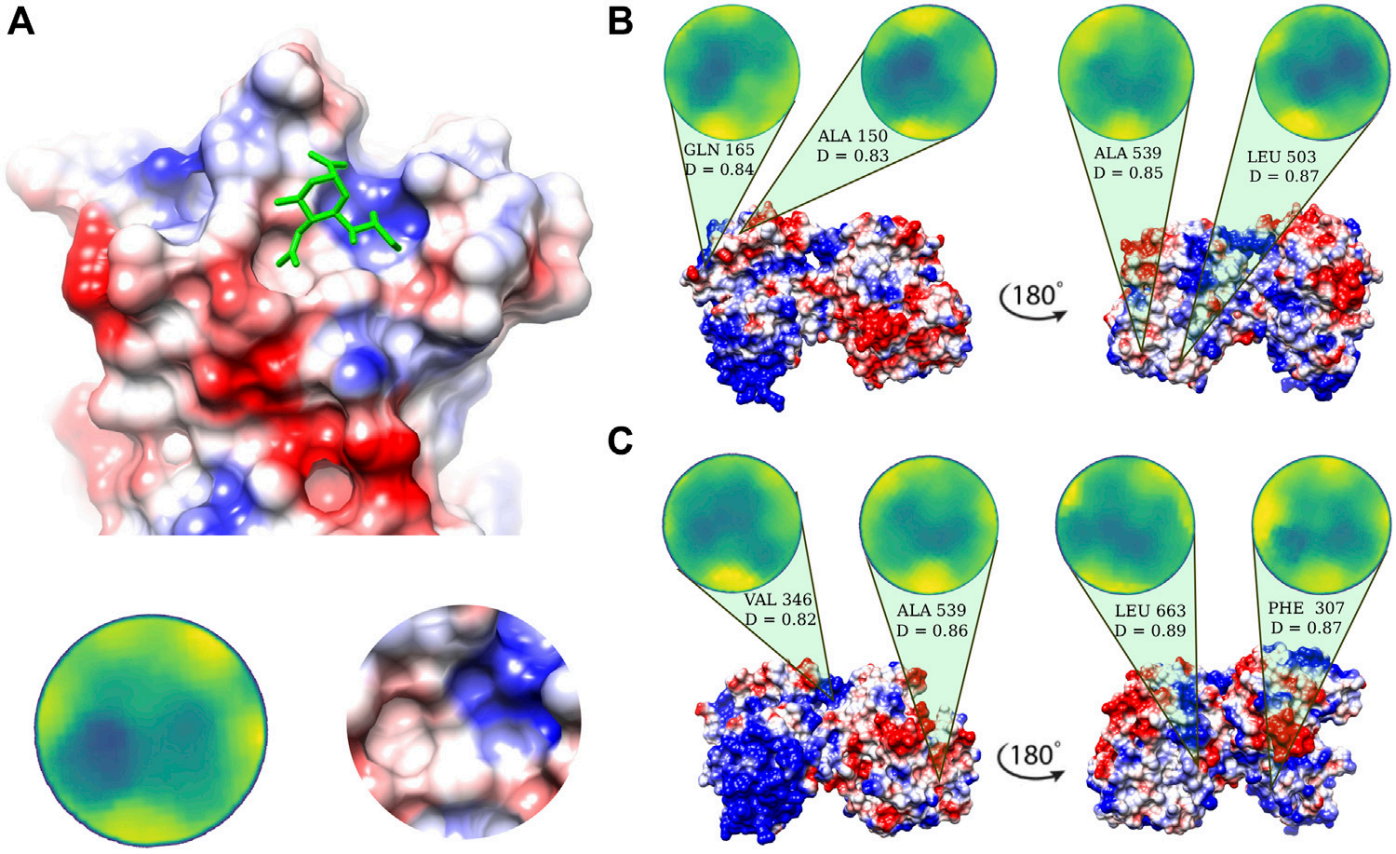
Putative sialic-binding regions on human lactoferrin. **(A)** Cartoon representation of the sialic-acid binding region of MERS-CoV (PDB id: 6Q04) and its representation in the Zernike disk (left) and local Coulombic surface (right). **(B)** Four most complementar regions on holo human lactoferrin (PDB id: 1LFG) obtained comparing the lactoferrin molecular surface with the sialic acid binding region of MERS-CoV. Below each disk, the central residue of the patch is reported, together with the Euclidean distance, *D*, (see Eq. 6) between the Zernike descriptors of the patch with respect to the one displayed in panel **A**). **(C)** Same as in **(B)** but for the apo human lactoferrin (PDB id: 1CB6). Molecular surfaces are colored according to the Coulombic potential.

Possible binding regions for sialic acid, the terminal molecule of the SIA chains on human lactoferrin are investigated on the basis of the procedure described in Milanetti et al. (2020a), i.e. we select the portion of the molecular surface of the MERS-CoV spike protein in interaction with sialic acid, experimentally solved in Park et al. (2019) (Figure 2A), and we search for similar patches on the Lf molecular surface. Within the same strategy, we search for similar patches on the surface of human lactoferrin (see *Methods* for more details).

In the 2D Zernike framework, the geometrical shape of a protein surface patch is compactly summarized in a set of ordered numerical descriptors, whose number - 121 in our case - modulate the detail of the description. Dealing with ordered numerical descriptors, the comparison between different protein patches can be performed with a Euclidean distance. Figures 2B,C show the four most geometrically similar patches identified in the Apo and Holo form of Lf. An *a posteriori* check of the electrostatic potential on the patches, allows us to select only some of the possible solutions identified based on the shape comparison analysis, i.e. the ones having also a similar electrostatic surface with the SIA binding site on the MERS-CoV spike.

The region on the Lf surface identified as the most similar to the MERS region interacting with sialic acid, both in shape and in electrostatics, is the one centered on VAL 346.

### Interaction with ACE2

**FIGURE 3:**
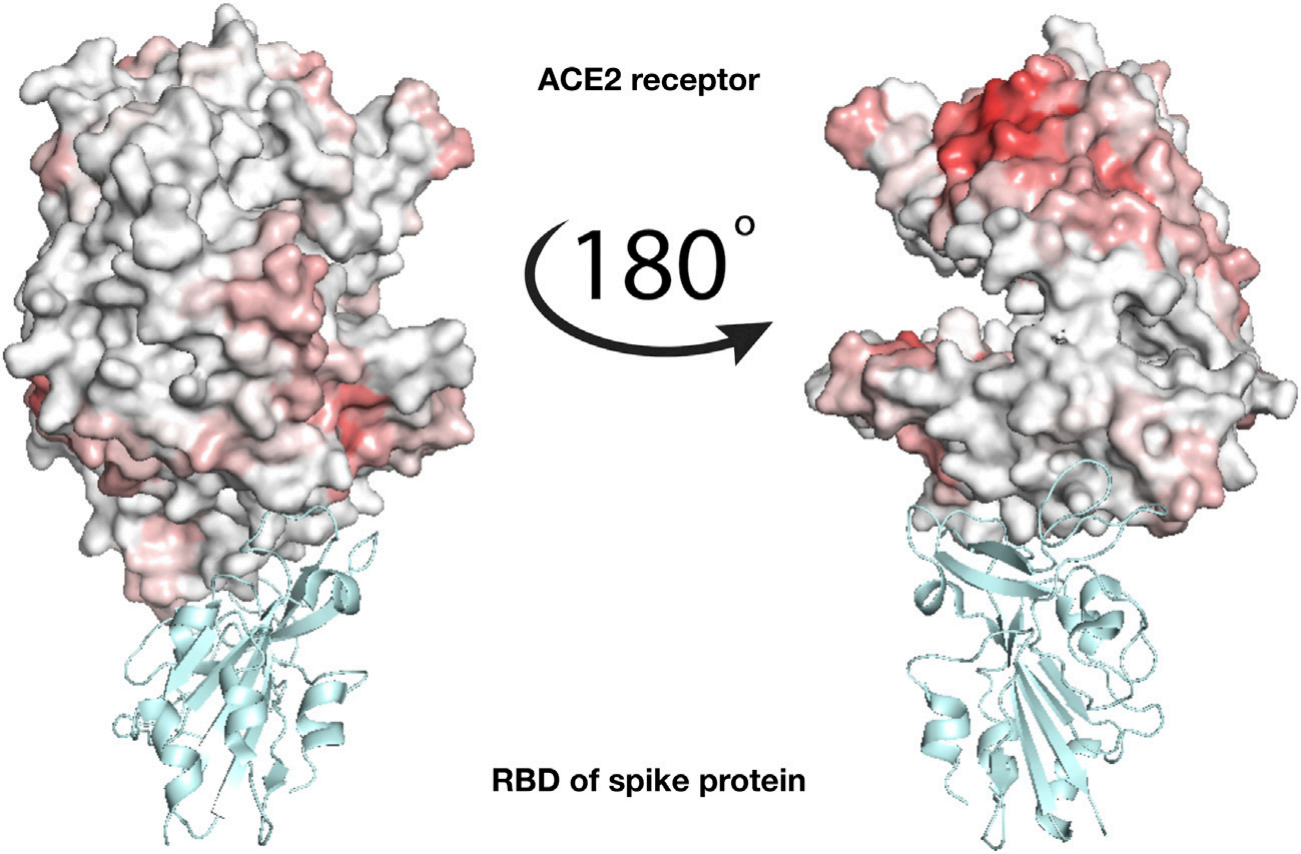
Analysis of the binding between lactoferrin and the ACE2 receptor. Molecular representation of the experimentally solved complex of the ACE2 receptor with SARS-CoV-2 spike receptor-binding domain, RBD (PDB id: 6m17). ACE2 molecular surface is colored according to its binding propensity to bind lactoferrin regions. Dark red indicates high binding propensity while white means no interaction. The spike RBD is colored in cyan.

To check whether the action of lactoferrin can be ascribed to a competition with the virion spike proteins in binding directly the ACE2 receptor (Figure 1), we performed a blind search of the molecular surfaces of both ACE2 and human Lf to identify possible binding regions having a meaningful shape complementarity. Under this hypothesis, if the interaction between the Lf and the ACE2 receptor occurs, Lf could hinder the molecular binding between the spike protein of SARS-CoV-2 and the corresponding ACE2 receptor. Figure 3 shows the molecular surface of the ACE2 receptor colored according to its propensity to bind regions of the Lf protein. The redder the region, the greater the shape complementarity between that region and another one found on the surface of the putative molecular partner, i.e. holo lactoferrin. As one can see from Figure 3, a complementary region is indeed present, however, it is located far from the binding site of the spike (grey in the figure) and in a part of ACE2 that looks toward the membrane. To better visualize the result, we have represented two points of view of the binding between spike and ACE2, one rotated 180^°^ with respect to the other. On the other hand, we can see that the ACE2 region interacting with the SARS-CoV-2 spike protein has no low shape complementarity with Lf regions.

### Interaction with Virion Membrane Proteins

A third possible mechanism at the basis of the observed antiviral activity of Lf could be ascribed to a direct interaction with the membrane proteins present on the virion envelope. In particular, SARS-CoV-2 presents three different kinds of proteins on its membrane, i.e. S, M, and E proteins Bianchi et al. (2020) and Seah et al. (2020). While the 3D structure of the S protein has been determined - even if some loop regions in the S1 sub-unit are not solved - unfortunately, no structures are available for the E and M proteins. Thus, the molecular surfaces of Lf were compared with those of the three proteins in order to check whether an interaction with lactoferrin is possible. For this analysis, we adopted the same computational procedure used in the previous paragraph.

For both S, M, and E proteins, we sample their whole molecular surface and compared all the possible patches with those of Lf. In this way, all molecular surfaces, both membrane proteins, and Lf ones are colored according to the corresponding binding propensity.

E and M presented a possible region of interactions located in the intra-membrane region (data not shown). The most robust and relevant result of this analysis regards the compatibility between the spike and lactoferrin. According to our findings, Lf presents two regions of high complementarity with one portion of the C-terminal domain of the spike S1 subunit and another located in the N-terminal part.

**FIGURE 4:**
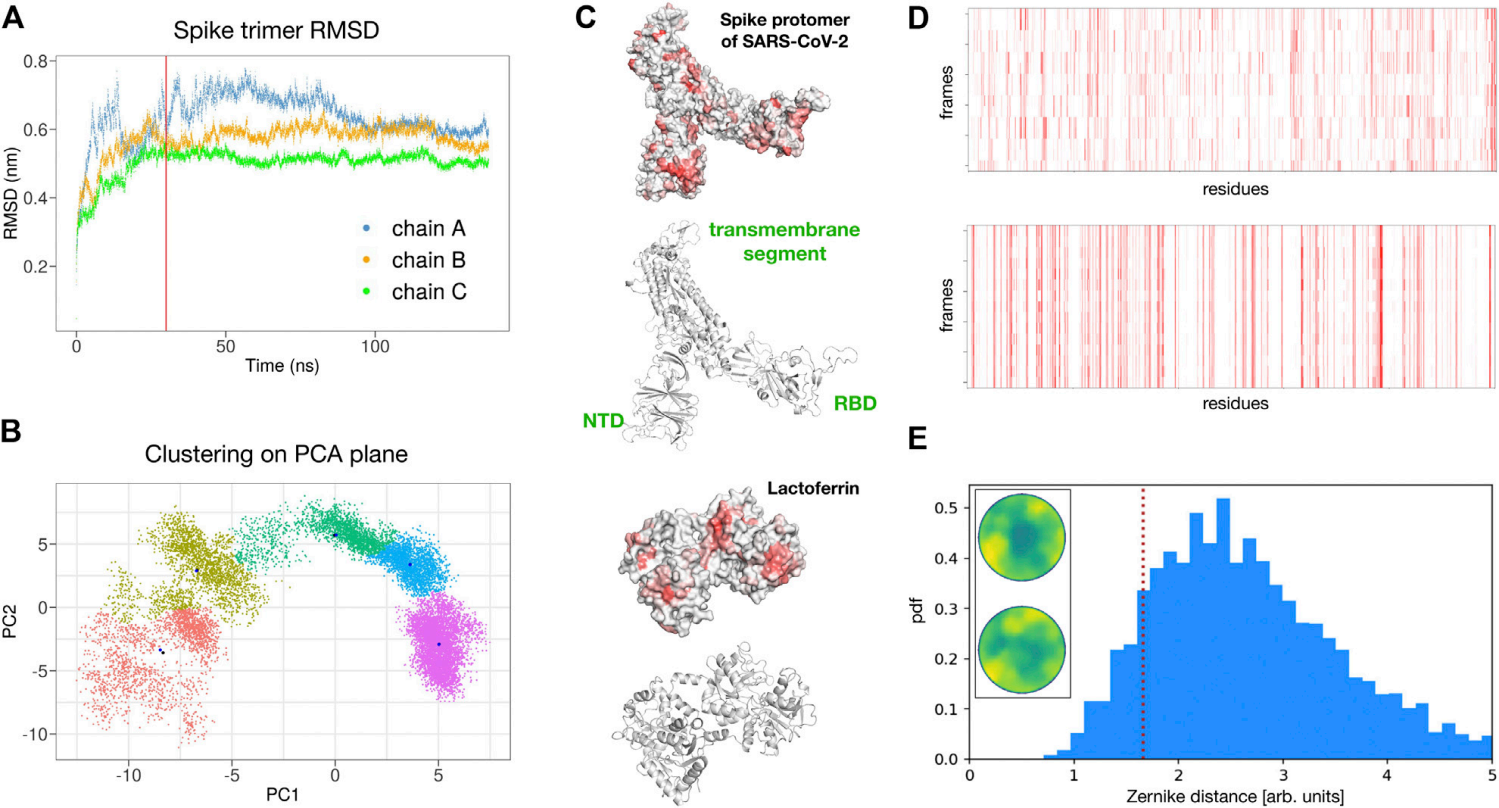
Possible interaction between SARS-CoV-2 spike protein and human lactoferrin. **(A)** Root mean square displacement as a function of time of the SARS-CoV-2 spike trimer as provided by the molecular dynamics simulation. **(B)** Clustering analysis of the spike trimer in the plane of the two principal components of a PCA analysis over the MD configurations. Five regions of major variability are identified. **(C)** Surface and ribbon representations of the spike protomer (chain A) and human lactoferrin. Regions of the molecular surfaces are colored from white to red according to their increasing shape complementarity with the partner. **(D)** Binding propensity computed from the Zernike descriptors between the 15 most variable conformations of the three chains of the SARS-CoV-2 Spike protein and human lactoferrin. Each residue of the proteins is colored from white to red according to its increasing shape complementarity with the partner. **(E)** Comparison between the complementarity score (red dashed line) of the best binding site (Zernike disks on the left) and the distribution of complementarity scores belonging to 4,600 binding regions of experimental complexes.

To test the reliability of the found signals, we performed a molecular dynamics simulation of the spike trimer (see *Methods* for details) and sampled five configurations for each of the three chains at equilibrium (see Figure 4A).

In particular, for each chain, we performed a Principal Component Analysis (PCA) on the frames of the dynamics and thus projected them on the plane identified by the two principal components. Upon clustering these points we obtain five subgroups. For each subgroup, we extract the centroidal configuration. Since distant points in the PCA plane correspond to the different 3D structure, picking one point from each identified cluster assured that the selected configurations have high structural differences between them in the explored configuration space (see Figure 4B).

Remarkably, repeating the blind search for complementarity regions on the 15 surfaces of the extracted spike monomers, we found conservation of the signal over the conformational noise. Figure 4C) shows the identified regions and their conservation in the different sampled frames.

Among the found regions, the one involving the spike C-terminal domain is the one with higher shape complementarity. In particular, according to our method, human lactoferrin could interact with the spike protein with the surface region centered in residue ALA 539. Alternatively, the spike protein may interact with the Lf protein using a molecular patch centered in the residue PHE 490. Since the spike is usually heavily glycosylated (see for example Casalino et al. (2020) for details), we checked whether the identified region could be hidden by glycans. The closest glycosylation site (ASN 343) has a distance of 34 Å from the found region, thus the interaction with Lt is not expected to resent from the glycan shield. furthermore, it must be pointed out that the complementarity achieved by the identified patches is comparable with those of experimentally solved complexes. Indeed, analyzing the shape complementarity of over 4,600 x-ray protein-protein complexes (see *Methods* for details), we have the distribution shown in Figure 4D, where the lower the distance the higher the complementarity of the binding region. The red dotted line shows the complementarity found between the spike and Lt. As it is evident the proposed patch is characterized by values of shape complementarity typical of experimental complexes.

Finally, to further support this result with an independent and external methodology to our approach, we performed a completely blind molecular docking analysis between the spike protein and the Lf protein. To this end, the Zdock server was used as a state of the art of molecular docking software Chen et al. (2003). As per default settings, only the first 10 docking poses have been selected and the predicted contacts analyzed. In particular, five out of ten poses show bindings involving the spike region involve the region we found with our protocol when the residues are defined in contact if their C-alpha atoms have a distance less than 8 *Å*.

## Discussion and Conclusions

In the last decade, the Zernike formalism has been widely applied for the characterization of molecular surfaces Venkatraman et al. (2009), Kihara et al. (2011), Di Rienzo et al. (2017), Daberdaku and Ferrari (2019), and Di Rienzo et al. (2020).

Very recently, we developed a new representation, based on the 2D Zernike polynomials, which allows an extremely efficient, fast, and completely unsupervised description of the local geometrical shape, allowing for easy comparison between different regions of molecules. Through this compact description, it is possible both to analyze the similarity between two different regions - suggesting, for example, a similar ligand for binding regions - and to study the complementarity between interacting surfaces Milanetti et al. (2020a).

Here, we used our novel method to shed some light on the molecular mechanisms that may support the antiviral action of human lactoferrin against SARS-CoV-2 infection. In particular, we focused on the early stages of the infection, i.e. the attachment and entry of the virus to the host cell, when lactoferrin can interfere with the virus-host interaction without the need to be internalized in the cell. We thus tried to establish whether lactoferrin could compete with the virus in binding to sialic acid, the sticky end of sialoside chains, which has been suggested to mediate the attachment of SARS-CoV-2 to the host cell. Interestingly, comparing the binding region of sialic acid in the MERS-CoV coronavirus with patches on the lactoferrin surface, we found possible spots on both apo and holo forms of Lf, which could compete in forming low affinity but high avidity interactions.

We then proceeded to test the hypothesis of an interaction between lactoferrin and the primary SARS-CoV-2 protein receptor, ACE2. A blind search for complementarity regions highlighted a hot-spot in a region that in physiological conditions is oriented toward the membrane, while no significative complementarity is present in the ACE2 region involved in the interaction with the virus spike protein. At last, we analyzed the three membrane proteins on the virus envelope, i.e. the E, M, and S ones. Similarly to ACE-2, both E and M presented possible interacting regions in portions of the surface, that are buried in the virion membrane under normal conditions (data not shown). On the other hand, the spike protein showed two main hot spots, one in the N-terminal domain of the S1 subunit and another in the C-terminal one. Those two regions are robust to molecular noise, as the signal endures using different configurations sampled from a molecular dynamics simulation and each of the three chains of the trimer. Notably, the most complementary region is the one in the C-terminal region, the one involved in the spike-ACE2 interaction. Notably, this region is far from known spike glycosylation sites and therefore even more available for the binding. This aspect is quite important if we consider that the spike is usually heavily glycosylated, with a large fraction of the protein surface buried under a glycan shield Casalino et al. (2020). Thus our finding suggests a possible competition between ACE2 and lactoferrin for the binding of the SARS-CoV-2 spike, which may explain the observed antiviral action.

## Materials and Methods

### Datasets

The protein, whose structures are analyzed in this paper are:

• Human lactoferrin, in the apo (PDB id: 1CB6) and holo (PDB id: 1LFG) forms.

• ACE2, in its apo state (PDB id: 1R42).

• Unbound SARS-CoV-2 spike protein: modeled by I-TASSER server Roy et al. (2010).

In a nutshell, the used server works in three steps: 1) starting from the submitted amino acid sequence, it first tries to retrieve template proteins of similar folds (or super-secondary structures) from the PDB library. Then, 2) the continuous fragments excised from the PDB templates are reassembled into full-length models. In cases where no appropriate template is identified, I-TASSER builds the whole structure by ab initio modeling. Finally, 3) the algorithm refines the models by removing steric clashes as well as checking the global topology of the found models, optimizing the hydrogen-bonding network.

The 10 structural analogs (as identified by TM-align Zhang and Skolnick (2005)) found by I-tasser are PDB id: 5X58, 6NZK, 6NB3, 3JCL, 5I08, 6CV0, 5SZS, 5WRG , 6UTK, 6B7N. The first (out of five) model returned by the server has been used as a starting conformation for the molecular dynamics simulation. We note that the obtained model has an average RMSD of ∼1.2Å with recently published experimental structures (see e.g. PDB id: 6VSB, 6VXX, and 6VYB).

• SARS-CoV-2 M protein, modeled using I-Tasser Roy et al. (2010). The 10 structural analogs (as identified by TM-align Zhang and Skolnick (2005)) are PDB id: 3W6Q, 4OUA, 4HE8, 4YU5, 1JS8, 3RKO, 2Y9W, 1TJ7, 2RF7, 4BED. The first (out of five) model returned by the server has been used for the analyses.

Additionally, we compared the structure of the M protein provided by I-tasser, with the one recently predicted by AlphaFold Senior et al. (2020). We found that while almost all the secondary structures are correctly predicted by both algorithms, the overall fold of the protein is better reproduced in the AlphaFold model, even if it lacks the first 10 residues of the N-terminal domain, which is the part of the M protein that protrudes from the virion membrane Thomas (2020).

• SARS-CoV-2 E protein, modeled using I-Tasser Roy et al. (2010). Here, we directly provided the experimentally solved SARS-CoV E protein (PDB id: 2mm4) as a template for homology modeling. The first (out of five) model returned by the server has been used for the analyses. Notably, comparing the model structure with the recently solved trans-membrane region of SARS-CoV-2 E protein (PDB id: 7k3g), we obtained an RMSD of 2.6 A.

To set a reference for the measured complementarities, a dataset of protein-protein complexes experimentally solved in x-ray crystallography is taken from Gainza et al. (2020). We only selected pair interactions regarding chains with more than 50 residues. The Protein-Protein dataset is therefore composed of 4605 complexes. For each complex, the binding region is identified as the portions of the two protein molecular surfaces distant less than 3 *Å*.

### Computation of Molecular Surfaces

For each protein of the dataset (x-ray structure in PDB format Berman et al. (2003)), we use DMS Richards (1977) to compute the solvent-accessible surface, using a density of 5 points per ${Å}^{2}$ and a water probe radius of 1.4 *Å*. The unit normals vector, for each point of the surface, was calculated using the flag −n.

### Patch Definition and Complementarity Evaluation

A molecular surface is represented by a set of points in the three-dimensional space. We define a surface patch, as the group of points that fall within a sphere of radius $R_{s}=6Å$, centered on one point of the surface. Once the patch is selected,

• we fit a plane that passes through the points and reorient the patch in such a way to have the z-axis perpendicular to the plane and going through the center of the plane.

• we define the angle θ as the largest angle between the perpendicular axis and a secant connecting a given point *C* on the z-axis to any point of the patch. *C* is then set in order that $\theta =45^{\circ }$. *r* is the distance between *C* and a surface point.

• build a square grid and associate each pixel with the mean *r* of the points inside it. This 2D function can be expanded on the basis of the Zernike polynomials (see next section).

Once a patch is represented in term of its Zernike descriptors, the similarity between that patch and another one can be simply measured as the Euclidean distance between the invariant vectors. The relative orientation of the patches before the projection in the unitary circle must be considered. In fact, if we search for similar regions we must compare patches that have the same orientation once projected in the 2D plane, i.e. the solvent-exposed part of the surface must be oriented in the same direction for both patches, for example as the positive z-axis. If instead, we want to assess the complementarity between two patches, we must orient the patches contrariwise, i.e. one patch with the solvent-exposed part toward the positive z-axis (“up”) and the other toward the negative z-axis (“down”).

### 2D Zernike Polynomials and Invariants

Each function of two variables, f(r,ϕ) (polar coordinates) defined inside the region r<1 (unitary circle), can be decomposed in the Zernike basis as

$$f\left(r,\phi \right)=\sum_{n=0}^{\infty }\sum_{m=0}^{m=n}c_{nm}Z_{nm}$$ (1)

with

$$c_{nm}=\frac{\left(n+1\right)}{\pi }\langle Z_{nm}|f\rangle =\frac{\left(n+1\right)}{\pi }{\int^{\text{​}}}_{0}^{1}drr{\int^{\text{​}}}_{0}^{2\pi }d\phi Z_{nm}^{\text{*}}\left(r,\phi \right)f\left(r,\phi \right).$$ (2)

being the expansion coefficients, while the complex functions, $Z_{nm}\left(r,\phi \right)$ are the Zernike polynomials. Each polynomial is composed by a radial and an angular part,

$$Z_{nm}=R_{nm}\left(r\right)e^{im\phi }.$$ (3)

where the radial part for any *n* and *m*, is given by

$$R_{nm}\left(r\right)=\sum_{k=0}^{\frac{n-m}{2}}\frac{{\left(-1\right)}^{k}\left(n-k\right)!}{k!\left(\frac{n+m}{2}-k\right)!\left(\frac{n-m}{2}-k\right)!}r^{n-2k}$$ (4)

Since for each couple of polynomials, the following relation holds

$$\langle Z_{nm}|Z_{n\text{'}m\text{'}}\rangle =\frac{\pi }{\left(n+1\right)}\delta_{nn'}\delta_{mm'}$$ (5)

the complete set of polynomials forms a basis and knowing the set of complex coefficients, $\left\{c_{nm}\right\}$ allows for a univocal reconstruction of the original image (with a resolution that depends on the order of expansion, N=max(n)). Since the modulus of each coefficient ($z_{nm}=|c_{nm}|$) does not depend on the phase, i.e. it is invariant for rotations around the origin of the unitary circle, the shape similarity between two patches can be assessed by comparing the Zernike invariants of their associated 2D projections. In particular, we measured the similarity between patch *i* and *j* as the Euclidean distance between the invariant vectors, i.e.

$$d_{ij}=\sqrt{\sum_{k=1}^{M=121}{\left(z_{i}^{k}-z_{j}^{k}\right)}^{2}}$$ (6)

### Detection of Similar Regions

The search for possible regions of interaction with sialic acid on the lactoferrin surface has been performed similarly to what done in Milanetti et al. (2020a). First, we identified the interaction region of sialic acid on the MERS-CoV surface. Next, we isolated the surface patch selecting all surface points within a sphere of radius 6Å centered around residue I-132 of the N-terminal domain of MERS-CoV spike (see Figure 2A) and computed the corresponding Zernike descriptors. Then, we repeated a similar procedure using each point of the lactoferrin surfaces (both apo and holo forms) as centers for the sphere. Finally, we evaluate the Euclidean distance between the Zernike descriptors of the MERS-CoV disk (reference patch) and those associated with the Lt surface points. All patches were studied in the “up” orientation (see previous sections). In Figures 2B,C), we show the four regions having lower Euclidean distance with respect to the reference patch.

### Detection of Complementar Regions

To evaluate whether two proteins present regions with high shape complementarity (and thus of possible interaction), we first computed the Zernike descriptors of the patches centered in all the points of the two surfaces, choosing for one protein the “up” orientation and for the other the “down” orientation. Next, we computed the Euclidean distance between all possible couples of patches among the two proteins. We thus associate to each point of the two surfaces the minimum distance value observed - *the binding propensity* - between the considered point and all points of the other surface. After all surface points are associated with their binding propensity, we performed a smoothing process to highlight the signal in specif regions characterized mostly by low distance values. In this process each point is associated with the mean value of the points in its neighborhood: the basic idea is that the interacting region should be made up mostly of elements with high complementarity and therefore a high average value of binding propensity values (see Milanetti et al. (2020b, a) for further details). For both the patch definition and the smoothing process we adopted a sphere radius of 6 *Å*.

### Molecular Dynamics Simulations

The starting structure of the SARS-CoV-2 spike trimeric complex was taken from the model structure proposed by the I-Tasser server Roy et al. (2010). All steps of the simulation were performed using Gromacs 2019.3 Spoel et al. (2005). Topologies of the system were built using the CHARMM-27 force field Brooks et al. (2009). The protein was placed in a dodecahedric simulative box, with periodic boundary conditions, filled with 131793 TIP3P water molecules Jorgensen et al. (1983). We checked that each atom of the trimer was at least at a distance of 1.1 nm from the box borders. The addition of three sodium counterions rendered the systems electroneutral. The final system, consisting of 448,572 atoms, was first minimized with 2,064 steps of steepest descent. Relaxation of water molecules and thermalization of the system in NVT and NPT environments were run each for 0.1 at 2 fs time-step. The temperature was kept constant at 300 K with v-rescale algorithm Bussi et al. (2007); the final pressure was fixed at one bar with the Parrinello-Rahman algorithm Parrinello and Rahman (1980) which guarantees a water density of 1,004 $\text{kg}/{\text{m}}^{3}$, close to the experimental value. LINCS algorithm Hess et al. (1997) was used to constraint h-bonds.

Finally, the systems were simulated with a 2 fs time-step for 140 ns in periodic boundary conditions, using a cut-off of 12 *Å* for the evaluation of short-range non-bonded interactions and the Particle Mesh Ewald method Cheatham et al. (1995) for the long-range electrostatic interactions.

## Data Availability

The raw data supporting the conclusions of this article will be made available by the authors, without undue reservation.
